# Fixation duration on natural scenes is explained by memory encoding not processing demand

**DOI:** 10.1038/s41593-026-02285-1

**Published:** 2026-05-25

**Authors:** Philip Sulewski, Carmen Amme, Martin N. Hebart, Peter König, Tim C. Kietzmann

**Affiliations:** 1 Institute of Cognitive Science, Osnabrück University, Osnabrück, Germany; 2 Vision and Computational Cognition Group, Max Planck Institute for Human Cognitive and Brain Sciences, Leipzig, Germany; 3 Department of Medicine, Justus Liebig University Giessen, Giessen, Germany; 4 Center for Mind, Brain and Behavior, Universities of Marburg, Giessen and Darmstadt, Marburg, Germany; 5 Department of Neurophysiology and Pathophysiology, Center of Experimental Medicine, University Medical Center Hamburg-Eppendorf, Hamburg, Germany

**Keywords:** Learning and memory, Decision, Visual system, Attention, Computational neuroscience

## Abstract

Before each of around 200,000 eye movements we make each day, the brain decides how long to fixate before shifting gaze to new information. Here we investigate this process using a large-scale scene-viewing experiment (4,080 natural scenes, five participants) that combines magnetoencephalography, eye tracking and a semantic captioning task. Using multivariate analysis of magnetoencephalography source-space patterns, behavioral analyses and artificial neural network (ANN) modeling, we show that longer fixations do not reflect prolonged visual processing but relate to downstream memory encoding. First, temporal variability of ventral stream representational dynamics did not explain variability in fixation duration. Second, fixation durations were anticorrelated with ANN-estimated patch classification difficulty. Third, fixation durations correlate positively with ANN-predicted patch memorability and caption-inclusion and co-occur with increased theta–gamma phase–amplitude coupling, particularly in frontal and hippocampal regions. These results indicate that eye-movement timing decisions are shaped by memory-encoding demands rather than by perceptual processing limits.

## Abstract (web summary)

By combining magnetoencephalography and eye tracking, this study sheds light on why people fixate on some parts of natural scenes longer than others. Rather than visual complexity, fixation durations are affected by memory encoding.

## Main

Natural vision continuously involves deciding between maintaining our current fixation or redirecting our gaze to explore new visual information. Considering the high information density and dynamic changes of the natural world, a fast sampling of varied information seems preferable. However, our eyes remain at some locations for more than 500 ms, whereas others are visited for less than 150 ms (refs. ^1,2^), a striking delay given the brain’s remarkably fast processing speeds^3,4^. This large variation raises a fundamental question about the brain’s underlying computational strategy.

Although previous research has described correlations between fixation durations and local image features (for example, local contrast, edge density), task demands and exploration sequence parameters^1,2,5–9^, our understanding of the underlying neural information processing remains limited. We narrow this gap by developing process-related predictors that test specific hypotheses about the neural information processing mechanisms that guide fixation timing.

A prevalent theory of why our eyes rest longer at some locations is based on the consideration that the brain may require varying amounts of time to extract information. Indeed, in the case of static vision in macaques, Kar et al.^10^ demonstrated the need for recurrent information processing in cases of challenging stimuli, indicating that more complex visual stimuli demand prolonged neural computational time. Analogously, recurrent artificial neural network (ANN) models were shown to align with human reaction times in an animacy classification task^11^. The corresponding processing-demand hypothesis expects longer fixation durations for local image features that are comparably challenging to recognize. On the neural level, this view proposes a delayed convergence of representational dynamics when fixating challenging targets compared to simpler ones.

However, an alternative account is also possible. Based on the observation that ventral stream representations exhibit only limited cross-fixation integration^12^, ventral stream information is largely overwritten as the eyes move on to a new location. Prolonged fixations could therefore reflect a strategic time allocation of the brain to actively stabilize the neural embedding of a fixated patch to support downstream processes, such as memory encoding, before allowing a disruption by the next saccade. We term this alternative account of fixation durations the memory-facilitation hypothesis.

**Fig. 1:**
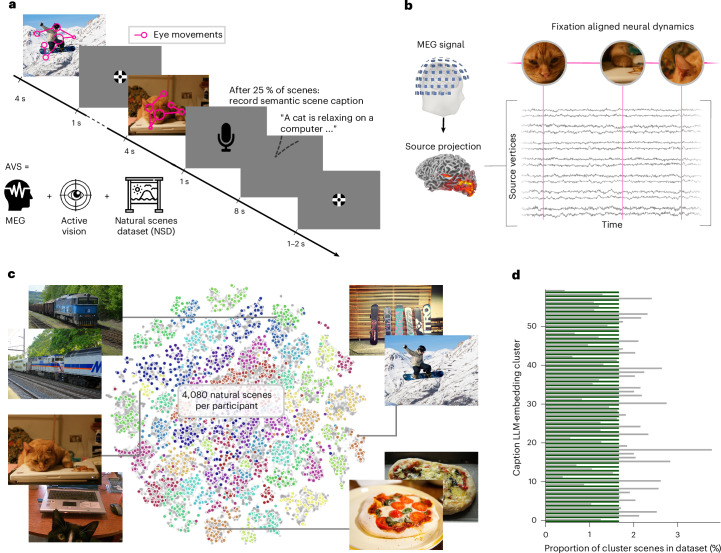
Understanding the neural dynamics of active scene viewing: the Active Visual Semantics dataset. **a**, Experimental design combining MEG recordings with natural scene exploration. Participants freely viewed 4,080 scenes (subsampled from the NSD dataset^13^) for 4 s each, with eye movements continuously recorded. After 25% of randomly selected trials, participants were tasked to verbally caption the scene. **b**, Analysis pipeline showing that MEG signals were source-projected and analyzed time-locked to fixation events to capture neural dynamics during natural viewing. **c**, Large language model (LLM) caption embedding space visualization (t-distributed stochastic neighbor embedding (t-SNE)) of the stimulus set. Each dot represents a scene, with colors indicating semantic clusters used for balanced sampling from the NSD scenes (gray dots). **d**, Distribution of scenes across 60 semantic clusters, demonstrating semantically balanced sampling. Green bars show the proportion of scenes per cluster in the Active Visual Semantics (AVS) dataset compared to the original NSD distribution (gray). Photos from the COCO image dataset/Flickr^51^. Source data

To differentiate between these two hypotheses, we collected a large-scale magnetoencephalography (MEG) dataset in which five participants actively explored 4,080 natural scenes (Fig. 1). The experiment consisted of a scene-captioning task during which participants freely explored each natural scene for 4 s, in 25% of cases followed by a request to provide a verbal semantic description (Fig. 1a). Throughout the experiment, we simultaneously recorded MEG signals and eye movements, allowing us to analyze the neural dynamics time-locked to fixations (Fig. 1b). The scenes were subsampled from the Natural Scenes Dataset (NSD^13^). To create a semantically diverse, yet balanced, stimulus set, we clustered the scenes’ caption embeddings into 60 semantic clusters and sampled uniformly across these (Fig. 1c,d; Methods).

This large-scale dataset of MEG and eye-tracking data collected during active vision on natural scenes enabled us to effectively differentiate between the two hypotheses. To test the processing-demand hypothesis, we estimated the dynamics of MEG source activation patterns as a function of fixation duration and derived image-complexity measurements from task-trained ANN models^11^. Memory-related downstream processing per fixation was derived based on explicit ratings of fixated targets being mentioned in the participants’ scene caption and ANN-based memorability scores^14^, estimating the likelihood of memorization of local scene patches. Finally, we searched the MEG source space for theta–gamma phase–amplitude coupling (PAC)^15,16^ as a signature of memory processing.

Our results provide evidence against the processing-demand hypothesis: neural pattern dynamics stabilized at consistent latencies regardless of fixation duration, and easier-to-classify patches received longer fixations. In contrast, targets later mentioned in scene captions and those with higher ANN-predicted memorability received longer fixations. Longer fixations also co-occurred with enhanced theta–gamma PAC in frontal and hippocampal regions. Together, these findings indicate that fixation timing reflects strategic time allocation for memory encoding rather than visual processing demands.

## Results

### Consistent latency of MEG pattern stabilization regardless of fixation duration

**Fig. 2:**
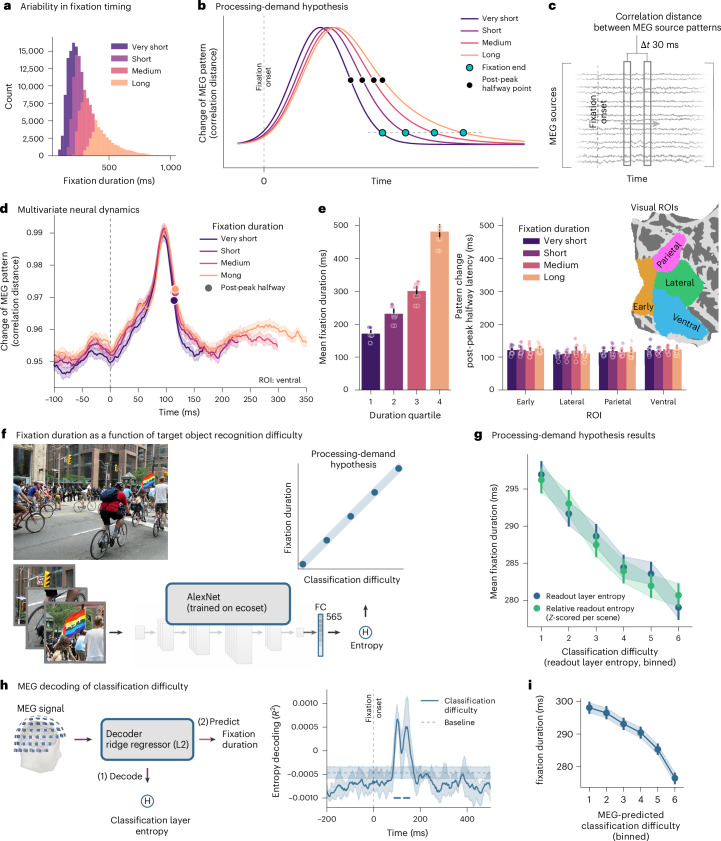
Visual processing demand does not translate to longer fixation durations. **a**, Distribution of fixation durations (all participants), sorted into duration quartiles (per participant). **b**, Hypothesized MEG pattern change over time for fixations of different durations, according to the processing-demand hypothesis. The stabilization of the MEG dynamics (quantified by the latency of the post-peak halfway points; black dots) is predicted to scale with fixation durations. **c**, Schematic illustration of the neural dynamics analysis approach. We computed the correlation distance between MEG source activation vectors (*p*) at consecutive time points separated by Δ*t* = 30 ms (D(t) = 1 − corr(*p*(*t*), *p*(*t* + Δ*t*)). **d**, Multivariate neural dynamics. Empirical MEG data showing ventral visual cortex dynamics during fixations of different durations. Despite variation in fixation length, the post-peak halfway points align across duration groups. Five participants (biological replicates; fixations within participants constitute technical replicates). Shaded areas: bootstrapped 95% confidence intervals (CIs) over fixation-level observations. **e**, left: mean fixation duration for each quartile group. Five participants, with individual data points overlaid. Right: post-peak halfway latency across different cortical regions, showing consistent timing regardless of fixation duration. Error bars: bootstrapped 95% CIs over two hemispheres per participant (*n* = 10). Group comparisons: two-sided linear mixed-effects model (duration quartile, ROI and hemisphere as fixed effects; participant as random intercept; all *P* > 0.25). **f**, Computational approach to quantify ease-of-recognition per fixation. We extracted crops from fixation locations and computed classification entropy (H) from an AlexNet^17^ model trained on ecoset^18^. The processing-demand hypothesis predicts longer fixation durations for challenging targets. **g**, Processing-demand hypothesis results. Mean fixation duration decreases as classification entropy increases, indicating longer fixations for easier-to-recognize content. The effect is observed for both absolute entropy (blue) and relative entropy (*Z*-scored per scene; green). Total of 324,522 fixations binned into six quantiles for each of five participants. Error bars: bootstrapped 95% CIs. **h**, MEG decoding of classification difficulty. Left: gradiometers were used to decode AlexNet classification layer entropy (H; 1). This decoder was used to subsequently predict the fixation durations from the decoded classification difficulty (2). Right: MEG decoding profile (solid blue line) compared to baseline (dashed blue line). Shaded regions show 95% CI. **i**, Higher decoded entropy predicts shorter fixations, providing neural validation of the inverse relationship observed with AlexNet-derived entropy (**g**). Total of 178,256 fixations at peak decoding time, binned into six quantiles per participant. Error bars: bootstrapped 95% CIs. Photo from the COCO image dataset/Flickr^51^. Source data

First, we examined whether different temporal dynamics of neural activity patterns could explain differences in fixation durations (Fig. 2a,b). For each visual region of interest (ROI), we computed the correlation distance between consecutive activation vectors with a moving 30-ms temporal gap. This measure quantified how neural activity patterns changed over time (Fig. 2c). Notably, the point of neural pattern stabilization—characterized as the halfway point of the falling flank following the main wave of pattern change—occurred at similar time points, regardless of later fixation duration (Fig. 2d) for all visual regions tested, including early visual, ventral, lateral and parietal regions. (Fig. 2e). A linear mixed-effects model with random intercepts for participants and controlling for hemisphere and ROI revealed that pattern stabilization timing was largely independent of fixation duration (all *P* > 0.25).

The observation that the mean latency of halfway points per duration quartile did not scale with mean fixation duration per quartile indicates that neural representations reached a relatively stable state at consistent timing, independent of how long a fixation lasted. This finding contrasts with the processing-demand hypothesis, which predicts delayed stabilization for longer fixations requiring extended recurrent processing.

### Easier rather than challenging targets receive longer fixations

To further examine the processing-demand hypothesis, we analyzed how the classification difficulty of fixated image patches relates to fixation duration. To estimate classification difficulty, we computed the classification layer entropy of an AlexNet model^17^ trained on ecoset^18^, where lower entropy indicates higher classification confidence^11^ (Fig. 2f). To ensure that fixation-based crops were not out of distribution for the task-trained ANN, we trained the model on a modified version of ecoset, focusing on size-matched image segments containing the respective ecoset categories. In comparing classification entropy with fixation duration, we found a negative relationship, indicating that easier-to-classify patches are fixated longer (Fig. 2g, blue line). Linear mixed-effects models, on log-transformed, *Z*-scored entropy values, confirmed that higher classification entropy predicted shorter fixations (*β* = −5.44 ms, 95% CI: (−6.07, −4.81), *P* < 0.001; *n* = 162,020 fixations, five participants). Importantly, this effect was not driven by scene-level differences, as the relationship persisted when computing the relative (*Z*-scored) classification entropy across all patches visited within a given scene (*β* = −5.13 ms, 95% CI: (−5.76, −4.50), *P* < 0.001; Fig. 2g, green line). This latter analysis confirmed that the effects observed are context-sensitive while operating at the level of individual fixations.

To provide neural validation of the relationship between recognition difficulty and fixation duration, we used MEG signals to decode the AlexNet-derived classification entropy. We trained ridge regression decoders on fixation-aligned MEG activity to decode the classification layer entropy values of fixated patches (Fig. 2h). Decoding accuracy peaked at 114.4 ms (95% CI: (98.0, 130.8)) post-fixation (*R*^2^ = 0.001, 95% CI: (0.001, 0.002), *P* < 0.001), demonstrating that visual recognition difficulty is, albeit minimally, decodable from gradiometer patterns. Critically, when using this decoder to predict classification entropy from the gradiometer topographies, we observed higher MEG-predicted entropy to predict shorter fixations (*β* = −3.89 ms, 95% CI: (−4.68, −3.10), *P* < 0.001), in line with our earlier behavioral observations. The negative relationship between classification difficulty and fixation duration generalized across multiple convolutional neural network architectures (AlexNet, VGG16, Inception-v3, ResNet50; Supplementary Fig. 1), indicating robustness of this effect. Taken together, contrary to the processing-demand hypothesis, which predicts longer fixations for more challenging visual inputs, our results revealed the opposite pattern: fixation durations were longer for image patches deemed easy to recognize.

### Longer fixations predict downstream utilization of visual information

**Fig. 3:**
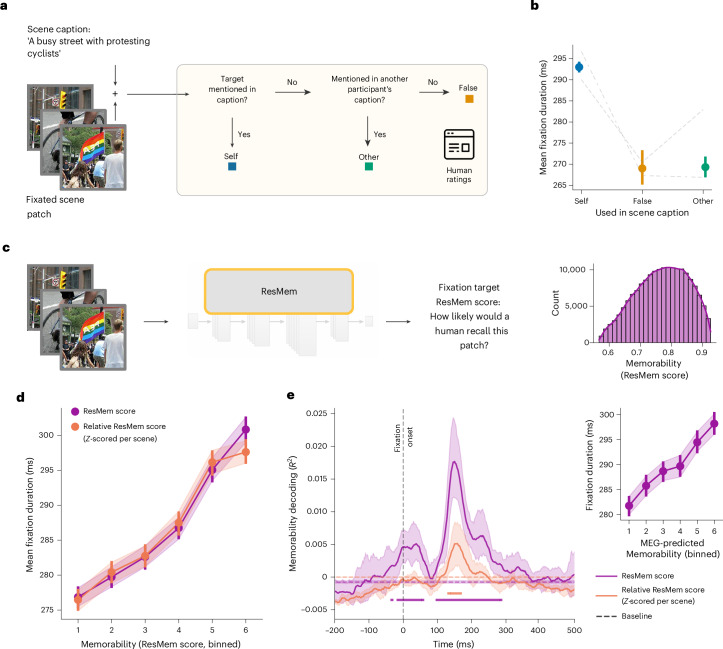
Memory-related downstream processing of visual information predicts fixation duration. **a**, Human ratings procedure for the relationship between fixation targets and scene captions. Each fixation target was rated as ‘self’ (mentioned in the participant’s caption), ‘other’ (mentioned in other participants’ captions but not their own) or ‘false’ (not mentioned in any caption). **b**, Mean fixation duration for each rating category. Fixation targets later referenced in the participant’s own caption received longer fixations than those not mentioned or mentioned only by other participants. Five participants; 86,738 fixations ratings (71,722 self, 11,417 other, 3,599 false). Error bars: bootstrapped 95% CIs. Gray lines indicate the two independent human raters. Group differences: two-sided linear mixed-effects models with participant as random intercept. **c**, Fixation target estimates of memory processing. We applied ResMem^14^ to each fixation location to predict how likely it was that humans would engage in memory processing when fixating. Histogram shows the distribution of memorability scores across all fixations and participants. **d**, Relationship between predicted memorability and fixation duration. Mean fixation duration increases with higher memorability scores, shown for both absolute ResMem scores (purple) and relative scores *Z*-scored per scene (orange). Shaded regions represent 95% CIs. **e**, MEG readout of memorability estimates. Left: decoding of ResMem scores from gradiometer activation patterns. Purple trace shows absolute ResMem score decoding, and the orange trace shows relative memorability (*Z*-scored per scene). Right*:* using the decoder to predict the memorability scores from the gradiometer patterns, the graph shows the positive relationship between MEG-predicted memorability and observed fixation duration. Five participants. Left: shaded areas show bootstrapped 95% CIs over participants; dashed lines show mean-prediction baselines. Right: 178,702 fixations (absolute)/178,545 fixations (relative) at peak decoding time points, binned into six quantiles per participant. Error bars: bootstrapped 99% CIs. Photo from the COCO image dataset/Flickr^51^. Source data

To test the memory-facilitation hypothesis, we asked whether fixations on objects that were later reported as part of the participants’ scene caption were fixated on longer compared to viewed but unreported objects. For each fixation target, two independent raters assessed whether it was later referenced in the participant’s scene description. For targets not mentioned by the participant, the raters additionally assessed whether other participants included them in their captions (Fig. 3a). We found the mean fixation duration to be longer for fixation targets later referenced in the participant’s own caption (293.0 ms, 95% CI: (292.0, 294.0)) compared to those not mentioned (269.0 ms, 95% CI: (265.2, 272.9) for targets never mentioned; 269.3 ms, 95% CI: (267.2, 271.4) for targets mentioned only by others; Fig. 3b). A mixed-effects model (*n* = 86,738 fixations) demonstrated that self-referenced targets had significantly longer fixations than both unreferenced targets (*β* = 25.7 ms, 95% CI: (21.3, 30.1), *P* < 0.001) and targets mentioned only by other participants (*β* = 21.1 ms, 95% CI: (18.5, 23.7), *P* < 0.001). Critically, the mean duration invested on targets mentioned only by others did not significantly differ from unreferenced targets (*β* = 4.7 ms, 95% CI: (−0.3, 9.6), *P* = 0.064), indicating that the effect was specific to individual participants’ subsequent use of visual information rather than visual target properties alone.

### Memorable fixation targets receive longer fixation durations

To expand our analyses beyond fixations on the 25% of scenes that were followed by the captioning task to all recorded fixations, we estimated patch memorability using an ANN trained to predict human memorability^14^ from large-scale behavioral visual memory datasets (LaMem^19^ and MemCat^20^; Fig. 3c). This model captures visual memory performance tested in time spans ranging from ∼27 s to ∼3.5 min, after 35–150 intervening images. The memorability estimates therefore allow for relating the observed fixation timing to estimates of memory processing for timescales that transcend the direct temporal coupling of scene exploration and subsequent captioning task.

In line with our previous results, we found the mean fixation duration to increase over six bins (sextiles) that capture increasing memorability estimates (Fig. 3d, magenta line). This relationship persisted for relative memorability estimates: that is, after normalizing the memorability estimates over the set of all targets visited in a scene (Fig. 3d, orange line). The latter indicates that the effects observed operate on a (context-sensitive) fixation-specific level, rather than reflecting overall scene memorability. Linear mixed-effects models with random intercepts for participants confirmed that both absolute memorability (*β* = 8.05 ms, 95% CI: (7.43, 8.66), *P* < 0.001) and relative memorability (*β* = 7.70 ms, 95% CI: (7.08, 8.31), *P* < 0.001) predicted longer fixations (*n* = 168,946 fixations, five participants). The positive relationship between memorability and fixation duration was robust across semantic categories, with significant effects for both animate and inanimate fixation targets (all *P* < 0.001; Supplementary Fig. 2). Taken together, targets predicted to be more memorable received longer fixations.

Previous work has shown that fixation duration also increases when the subsequent saccade targets a more distant scene location^2^. When including saccade amplitude in our model, the memorability effect remained robust (*β* = 8.07 ms, 95% CI (7.45, 8.69), *Z* = 25.50, *P* < 0.001), whereas saccade amplitude also predicted longer fixations (*β* = 2.39 ms, 95% CI (1.77, 3.01), *Z* = 7.54, *P* < 0.001). Notably, we observed a significant interaction between memorability and saccade amplitude (*β* = 2.74 ms, 95% CI (2.12, 3.36), *Z* = 8.66, *P* < 0.001), indicating that the memorability effect was stronger for fixations preceding larger saccades. This pattern suggests that memory encoding is strategically modulated by upcoming motor plans, supporting an active encoding process rather than passive processing bottlenecks.

A decoding analysis of the memorability, estimated across time, revealed that memorability could be successfully read out from gradiometer activation patterns. For absolute memorability, peak decoding accuracy (*R*^2^ = 0.020, 95% CI: (0.015, 0.026)) occurred at 148.8 ms (95% CI: (144.0, 154.0)) post-fixation-onset. For relative memorability, peak decoding accuracy (*R*^2^ = 0.007, 95% CI: (0.003, 0.010)) occurred at 122.0 ms (95% CI: (57.2, 158.4)) post-fixation-onset (*N* = 5 participants; Fig. 3e). Critically, to provide neural validation of the relationship between memorability and fixation duration, we used the output of the neural memorability decoder and related it to fixation durations. We observed higher MEG-predicted memorability for longer fixations (*β* = 5.62 ms, 95% CI: (5.01, 6.23), *P* < 0.001). These results reveal that memorability information relates to both fixation timing and neural activity patterns, suggesting that memory processes are relevant for active vision fixation timing.

### Theta–gamma coupling in neural dynamics during longer fixations

**Fig. 4:**
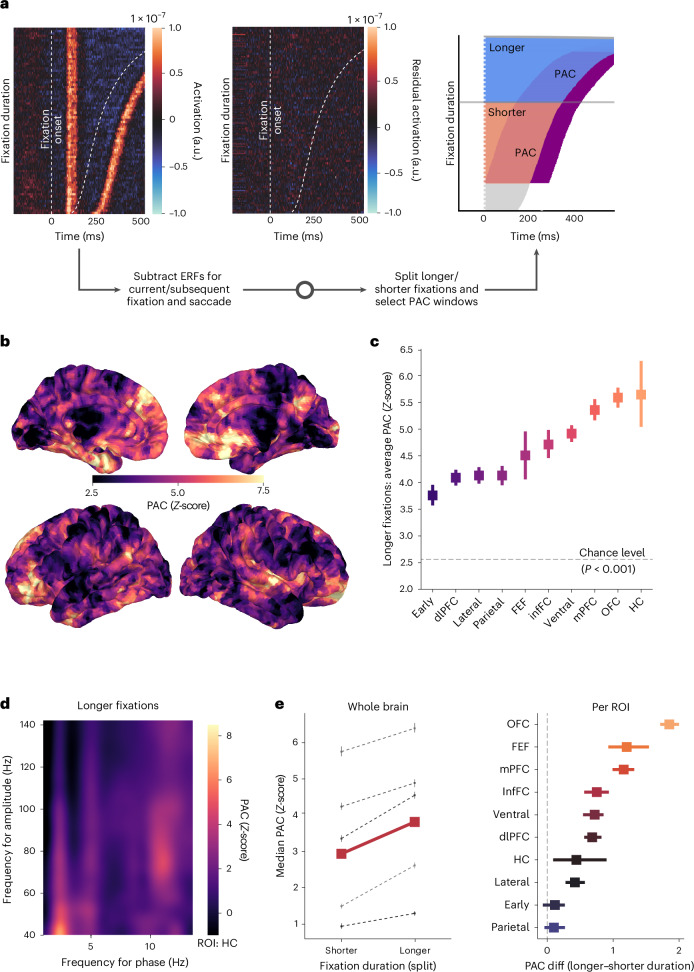
Neural signatures of memory-related processing. **a**, Illustration of the PAC analysis. Left: fixation-aligned MEG source activity (ROI: early visual cortex) showing fixation onset and offset activity. Median ERFs for 80 fixation duration sorted bins. Middle: after subtracting ERFs for the current and subsequent fixation and saccade (fourfold residuals). Right*:* PAC is computed in an offset-locked window during the final 250 ms before fixation offset. Fixations are split into longer (>300 ms) and shorter (<300 ms) duration categories, with PAC computed in equivalent offset-locked windows for both. **b**, Brain topography of theta–gamma PAC in longer fixations (averaged across five participants). Coupling strength (*Z*-score against surrogate baseline^15^) is displayed on cortical surfaces, showing widespread PAC across frontal, parietal and temporal regions. **c**, Average PAC strength per ROI during longer fixations. Error bars: bootstrapped 95% CIs over participant-hemisphere (*n* = 2 × 5) means. Dashed line: *Z* = 2.56 (*P* < 0.001). All reported ROIs showed significantly stronger coupling than early visual cortex (all *β* ≥ 0.23, all *P* < 0.05, uncorrected; two-sided linear mixed-effects model with participant as random intercept and early visual cortex as reference). **d**, Exemplary frequency-by-frequency PAC analysis for HC (left hemisphere), offering an extended window and finer resolution of the frequency bands contributing to the coupling. **e**, PAC contrast between longer and shorter fixations. Left*:* median PAC across the whole brain (all sources; subjects indicated as gray lines) for longer versus shorter fixations, showing enhanced coupling during longer fixations. Gray dashed lines show individual participants; error bars: bootstrapped 99% CIs over 81,958 source vertex PAC observations across five subjects with two duration groups each. Right*:* PAC difference (longer minus shorter) per ROI. Bootstrapped 95% CIs over participant-hemisphere medians (*n* = 2 × 5). Duration differences: two-sided linear mixed-effects models with participant as random intercept. dlPFC, dorsolateral prefrontal cortex; FEF, frontal eye field; HC, hippocampus; infFC, inferior frontal cortex; mPFC, medial prefrontal cortex; OFC, orbitofrontal cortex. Source data

Following our data-driven approach that identified the relation between fixation duration and memorability, we performed a targeted analysis in which we analyzed the fixation-aligned MEG data for PAC between theta and gamma oscillations, a neural signature of memory-related processing^21–27^. We focused our initial analyses on longer fixations lasting at least 300 ms and computed PAC within a window during the final 250 ms before fixation offset (Fig. 4a). This offset-locked approach captures neural activity preceding the decision to terminate a fixation.

To isolate oscillatory coupling from evoked responses, we subtracted the median event-related fields (ERFs) of the current saccade and fixation as well as the subsequent saccade and fixation (fourfold residuals; Fig. 4a). To ensure statistical robustness and control for spurious coupling, we employed the single-cut surrogate method^15^ that preserves the temporal structure of the data while disrupting the specific phase–amplitude relationships.

Contrasting our observed PAC measurements against this surrogate null distribution, we identified theta–gamma coupling across multiple cortical regions (Fig. 4b). In a widespread set of ROIs, we observed significant PAC in more sources per ROI than would be expected under chance (one-sample *t*-tests, FDR-corrected, all *P* < 0.05), demonstrating theta–gamma coupling above chance levels throughout the cortical hierarchy.

A mixed-effects model comparing PAC *Z*-scores across brain areas (with participant as random effect) revealed significantly stronger coupling compared to the early visual cortex (all *β* ≥ 0.23, all *P* < 0.05) in the lateral and parietal cortex, frontal eye field, inferior frontal cortex (infFC), ventral cortex, medial prefrontal cortex (mPFC), dorsolateral prefrontal cortex (dlPFC), orbitofrontal cortex (OFC) and hippocampus (HC) regions (Fig. 4c). To verify robustness of the PAC findings to the temporal window used for analysis, we repeated this analysis with an extended offset-locked window (final 350 ms before fixation end, with duration split at 350 ms; Supplementary Fig. 4). A separate frequency-by-frequency PAC analysis offered an extended window and a finer resolution of the bands involved in the observed PAC (Fig. 4d).

To directly test whether such memory-related coupling patterns are enhanced during longer fixations, we compared PAC strength between longer (>300 ms) and shorter (<300 ms) fixations, with both conditions analyzed using the same offset-locked window (final 250 ms before fixation offset; Fig. 4a). This comparison revealed significantly stronger theta–gamma coupling during longer compared to shorter fixations (Fig. 4e). A mixed-effects model testing duration differences (longer − shorter) per ROI indicated stronger PAC during longer fixations in frontal cortex: OFC (*β* = 1.99, 95% CI (1.39, 2.60), *P* < 0.001), frontal eye field (*β* = 1.14, 95% CI (0.43, 1.86), *P* = 0.002), mPFC (*β* = 0.97, 95% CI (0.37, 1.58), *P* = 0.002), infFC (*β* = 0.95, 95% CI (0.34, 1.57), *P* = 0.002) and dlPFC (*β* = 0.74, 95% CI (0.14, 1.33), *P* = 0.016), as well as in ventral cortex (*β* = 0.69, 95% CI (0.09, 1.29), *P* = 0.024). Together, these results demonstrate that longer fixations co-occur with enhanced neural signatures of memory encoding and maintenance, providing direct neural evidence for memory-related processing during extended viewing.

## Discussion

Using a large-scale MEG dataset in conjunction with eye tracking, behavioral analyses and computational modeling, we demonstrate that longer fixation durations are better explained by memory-related downstream processing rather than visual processing demands. Neural pattern dynamics stabilized at similar time points regardless of eventual fixation duration, and participants fixated longer on easier-to-classify rather than more complex image patches. This negative relationship between classification difficulty and fixation duration was preserved and validated in neural data when using a decoding model to predict the classification difficulty directly from the MEG signal.

This set of findings contrasts with prior evidence from macaque static viewing experiments in which challenging stimuli were shown to require prolonged recurrent processing (for example, ref. ^10^). Instead, our data suggest a more complex relationship during active vision on natural scenes, where further downstream processes, such as memory-related factors, exert substantial influence on fixation duration.

Four key findings support the involvement of memory-related processes as a driver of prolonged fixation durations: (1) objects later mentioned in scene captions received longer fixations; (2) targets that were predicted to have higher memorability via a deep network trained on human data consistently received longer fixations; (3) (relative) memorability of fixated patches was successfully decoded from MEG data, with higher decoded memorability predicting longer fixations; and (4) increased theta–gamma PAC was observed during longer fixations.

This oscillatory interaction, where gamma amplitude is modulated by theta phase, has been robustly linked to (visual) working memory encoding^21–29^. In this previous work, theta–gamma coupling was predominantly observed in frontal cortex, HC and parietal regions, aligning with the topographical pattern of theta–gamma coupling we observed.

Although PAC has been observed in contexts not limited to memory processes^26,30^, several observations support a mnemonic interpretation here. (1) The spatial distribution of enhanced PAC during longer fixations, strongest in frontal and hippocampal regions rather than visual cortex, aligns with memory networks^23,31,32^. Critically, (2) longer fixations are associated with both higher memorability and subsequent reporting in scene descriptions. The systematic modulation of PAC across fixation durations, combined with this behavioral link to memory outcomes, supports a memory-related interpretation in the present context. Being applied to fixation-aligned MEG data during natural scene exploration, the present PAC analysis operates on largely uncharted territory. For example, the ERFs associated with fixation onsets create transient spectral changes that may influence PAC estimates. Our fourfold ERF subtraction procedure, surrogate testing and duration contrast between longer versus shorter fixations thus provide important controls. However, future work will need to further explore the exact neural signatures of working memory during active vision.

Our findings of relatively stabilized visual information align with and extend observations by Xiao et al.^12^, who found that neural populations in the ventral stream process each fixation independently, observing no integration of information across saccades. Such fixation-specific processing in the ventral stream may explain the need for longer fixations on image regions that contain information needed for later downstream processes, as a fixation elsewhere would ‘flush’ the system with new information. Indeed, our data show that theta–gamma coupling occurs primarily in anterior frontal, orbitofrontal and hippocampal regions rather than in visual areas. This spatial dissociation suggests a division of labor: visual ventral stream areas process current fixations, whereas higher-order regions integrate, where needed, information across eye movements^33^, allowing for swift visual exploration while at the same time supporting an integrated understanding of the overall visual scene and contextualization of local information.

When exploring the visual environment, larger saccades involve greater spatial displacement, making it potentially more important to consolidate visual information before departing the current location. The finding that memorable content elicits proportionally longer fixations before larger saccades suggests that encoding is strategically modulated by upcoming motor plans. This pattern is consistent with an active, flexible encoding process rather than passive processing bottlenecks, where the system adaptively allocates encoding time based on both content value and upcoming displacement.

This perspective extends recent work that suggests a coordinated interplay between eye movements and memory processes. Previous studies have shown that saccade execution can negatively impact working memory retention^34,35^ while also being coordinated with hippocampal patterns that promote memory formation^36,37^. Additionally, individuals with higher working memory capacity show longer fixation durations during scene viewing^38^. This coordination between eye movements and memory processes is compatible with predictive coding frameworks^39^, in which eye movements could be described as experiments that test hypotheses about visual content^40,41^. Here memory representations guide predictions about what visual information to expect at different locations, enabling efficient sampling of informative regions and updating of internal scene models^42,43^.

Although our analysis reveals mechanistic insights into the decision process of how the brain decides when to sample information from a new location, the overall observed variability in fixation durations remains partially unexplained, likely reflecting a mixture of deterministic processes and stochastic influences^2,44,45^. Future work using models that incorporate semantic understanding^46^ or action-related features^47^ could provide additional insights into how the brain determines fixation allocation during natural vision.

Our dataset focuses on five participants, but this intensive within-subject design follows the approach of the NSD^13^ and other state-of-the art datasets that prioritize reliability and statistical power through extensive sampling of individual brains^48–50^. The perceptual and memory processes examined here are likely to reflect fundamental mechanisms that generalize across individuals, as suggested by the consistent effects observed in all participants. Nonetheless, future studies with larger and more diverse groups of participants will be useful to generalize these findings.

Our findings reveal that the substantial variability in fixation timing is primarily driven not by processing complexity but rather by downstream processing requirements. Visual representations stabilized at consistent time points regardless of fixation duration, contradicting processing-demand accounts. In contrast, memory-related theta–gamma coupling was stronger during longer compared to shorter fixations. These results establish active vision as memory-centric, where longer fixations reflect strategic temporal investment to stabilize neural codes before saccadic disruption. The brain therefore optimizes sampling duration based on information value, not computational complexity.

Our analyses of a large-scale MEG and eye-tracking dataset demonstrate that the case of active vision calls for a paradigm shift in visual neuroscience. In active vision, humans strategically allocate temporal resources based on downstream utility rather than immediate processing demands. This finding opens new avenues for understanding adaptive information gathering, with implications that extend from vision science to other sensory and cognitive domains. More broadly, these insights inform our understanding of how intelligent systems navigate exploration–exploitation trade-offs.

## Methods

### Data acquisition and preprocessing

#### Participants

Five healthy participants (three female, two male; mean age = 27.8 years, s.d. = 2.6) with normal or corrected-to-normal vision participated in the study. All participants provided written consent before participation and were financially compensated (€10 per hour for the magnetic resonance imaging (MRI) session, €9 per hour for MEG sessions plus a fixed €25 travel reimbursement per MEG session). The study was approved by the local ethics committee and conducted in accordance with the Declaration of Helsinki.

#### Stimuli and experiment

Participants explored 4,080 natural scenes from the NSD^13^, including all scenes from the shared-1000 subset. To ensure semantic variability, we implemented a systematic sampling approach based on scene caption semantic embeddings. We first generated semantic embeddings of the MS-COCO scene captions^51^ using a sentence-BERT model^52^, computing the average embedding across the five captions available for each scene. These embeddings were then clustered into 60 semantic clusters using *K*-means clustering. From each cluster, we uniformly sampled scenes to create a semantically balanced stimulus set while avoiding overrepresentation of any particular semantic category (Fig. 1c,d).

For visualization of the semantic embedding space, we applied *t*-distributed stochastic neighbor embedding dimensionality reduction with a perplexity of 30 and learning rate of 200 (Fig. 1c). Scenes were resized from their original MS-COCO dimensions to 947 pixels × 710 pixels using a centered crop-and-resize preprocessing that maintained aspect ratio while fitting the MEG laboratory display specifications (1,024 pixels × 768 pixels, utilizing 92.5% of the screen area).

Scenes were presented at a viewing distance of 70 cm on a screen measuring 41.6 cm × 31.2 cm, with the scenes covering 28.5° × 21.6° of the visual angle. Each scene was displayed for 4 s, followed by a variable 1-s to 2-s interstimulus interval. To encourage active engagement and semantic processing, participants performed a scene description task on 25% of randomly selected trials. During the task, the participants verbally described the content of the preceding scene within an 8-s response window. A blank gray screen was presented during this captioning task (Fig. 1a).

#### MEG acquisition

Brain activity was recorded using a 306-channel whole-head MEG system (Elekta Neuromag TRIUX, Elekta Oy) with 102 magnetometers and 204 planar gradiometers. Data were sampled at 1,000 Hz with an online filter of 0.1–330 Hz. Five head position indicator coils continuously tracked head movements. Head stabilization was achieved using individually fitted (based on three-dimensional head scans) foam casks filling out the MEG helmet, allowing for natural viewing behavior while minimizing head-movement artifacts.

#### Eye tracking

Eye movements were recorded with an EyeLink 1000 system (SR Research Ltd.) at a sampling rate of 1,000 Hz. The eye tracker was calibrated using a nine-point grid. The fixation cross preceding each scene was used as a drift correction. The next scene appeared only after successful drift correction. Gaze position data were aligned with the MEG recordings using triggers sent at the beginning of each trial. Saccades and fixations were detected using a velocity-based algorithm^53^. For each session, a velocity threshold was defined as five times the standard deviation of the sample-wise velocity. Samples with velocities exceeding this threshold were classified as saccades; all other samples were classified as fixations. Fixations shorter than 50 ms or longer than 1,000 ms were excluded from analysis.

#### MEG preprocessing

MEG data were processed using MNE-Python^54^. Temporal signal space separation with movement compensation was applied to suppress external noise (MaxFilter, Elekta Oy). Data were bandpass filtered between 0.2 Hz and 200 Hz and resampled to 500 Hz. Independent component analysis was applied to target ocular artifacts by removing independent components that correlated with the eye position. For source reconstruction, individual structural MRI scans were processed using FreeSurfer^55^ to obtain cortical surfaces.

#### Source reconstruction

The MEG (magnetometer and gradiometer) signal was reconstructed in source space using a linear constraint minimum variance (LCMV) beamformer approach. Individual structural MRI scans (3T) were processed using FreeSurfer to obtain cortical surfaces and generate individual anatomical models, and the forward model was computed using a three-layer boundary element method with source space constructed at 4,098 vertices per hemisphere using FreeSurfer ico4 surface decimation^55^. Head stabilization was achieved using individually fitted foam casks (based on three-dimensional head scans) that filled out the MEG helmet. Empty-room recordings acquired during each experimental session were used to estimate session-specific noise covariance matrices. For the LCMV beamformer, data covariance was computed from 350 randomly selected epochs concatenated across all 10 experimental sessions. LCMV spatial filters were computed per session using the session-specific noise covariance and concatenated data covariance, with regularization applied (reg = 0.05). The beamformer was configured with free orientation estimation and without weight normalization. Source activation was extracted for each epoch and vertex, with the final source estimate computed as the L2 norm of the three-dimensional source vector at each vertex. For group-level visualization, individual source estimates were morphed to the fsaverage template brain^55^.

#### ROI definition

All frontal regions and the HC were derived from the^56^ anatomical parcellation. The frontal regions (dlPFC, infFC, mPFC, OFC) were based on merging multiple Glasser regions specified as belonging to these structures. The early, lateral, ventral, and parietal regions were used as defined in NSD^13^ and correspond to anatomically defined visual processing streams, with the early visual cortex encompassing V1–V3 from the ref. ^57^ atlas, intermediate ROIs reflecting the posterior borders of each stream (ventral, hV4/IOG; lateral, LO1/LO2; parietal, V3A/V3B) and higher-level ROIs extending anteriorly along anatomical landmarks including the inferior temporal sulcus for ventral stream and intraparietal sulcus regions for parietal stream.

#### Epoch extraction

Data were epoched around both saccade and fixation onsets (−500 ms to 800 ms) based on the eye-tracking data. We matched the MEG and eye-tracking data streams at the starting time of each scene and defined the fixation/saccade based epochs accordingly (Fig. 1b).

### Analysis

#### MEG pattern change analysis

To quantify the temporal evolution of neural activation patterns during fixations, we computed correlation distances between consecutive source activation patterns separated by 30 ms within each ROI (Fig. 2b). A pattern stabilization over 30 ms is likely to indicate representational convergence rather than transient neural fluctuations. This choice is derived from prior work showing that functionally meaningful representational changes occur on this timescale^10^. Source-reconstructed data were first *Z*-scored per epoch over time to ensure comparable signal magnitudes across fixations and subjects:

$${X}_{\mathrm{z}}^{\,\left(i\right)}\left(v,t\right)=\frac{{X}^{\,\left(i\right)}\left(v,t\right)-{\mu}_{\mathrm{t}}^{\left(i\right)}}{{\sigma}_{\mathrm{t}}^{\left(i\right)}}$$

where ${X}^{\left(i\right)}\left(v,t\right)$ represents the activation at vertex *v* and time *t* for epoch *i*, and ${\mu }_{t}^{\left(i\right)}$ and ${\sigma }_{t}^{\left(i\right)}$ are the temporal mean and standard deviation for epoch *i* computed across all time points.

For each ROI containing *V* vertices, population vectors were constructed at each time point:

$${\bf{p}}\left(t\right)={\left[{X}_{z}\left({v}_{1},t\right),{X}_{z}\left({v}_{2},t\right),\ldots ,{X}_{z}\left({v}_{V},t\right)\right]}^{T}$$

The temporal dynamics were quantified by computing correlation distances between population vectors at time points *t* and *t* + Δ*t* using a one-sample stride:

$$D\left(t\right)=1-\mathrm{corr}\left({\bf{p}}\left(t\right),{\bf{p}}\left(t+\Delta t\right)\right)$$

where Δ*t* = 30 ms. This procedure was repeated for all time point pairs (*t,t* + Δ*t*), where *t* ranged from the start of the epoch to the final time point, allowing for the 30-ms offset, generating a time series of pattern change dynamics for each fixation epoch. Time points of the dynamics time series were masked when they exceeded the mean fixation duration for each duration quartile group. Pattern stabilization timing was approximated by identifying when neural dynamics reached the midpoint of their decline from peak to baseline following the main wave of pattern change. The halfway point was identified on median dynamics computed per fixation duration quartile, ROI, hemisphere and subject. The detection algorithm incorporated a tolerance-based approach to handle oscillations in the data and proceeded as follows:

1. Peak detection within the temporal window: Peaks were identified within 50-ms to 250-ms post-fixation-onset:
  $${t}_{\mathrm{peak}}={\mathrm{argmax}}_{t\in \left[50,250\right]}D\left(t\right)$$

(2) Halfway value computation: The target halfway value was calculated as
  $${D}_{\mathrm{halfway}}={D}_{\mathrm{min}}+\frac{D\left({t}_{\mathrm{peak}}\right)-{D}_{\mathrm{min}}}{2}$$

where *D*_min_ represents the minimum value across the entire dynamics time course.

(3) Search window definition: The search for the halfway point was constrained to 100 ms following the peak:
  $${T}_{\mathrm{search}}=\left[{t}_{\mathrm{peak}},{t}_{\mathrm{peak}}+100\right]\,\mathrm{ms}$$

(4) Tolerance-based halfway point detection: To handle oscillations in the neural data, points within a tolerance range of the halfway value were identified:
  $$\mathrm{tolerance}=\frac{\left(D\left({t}_{\mathrm{peak}}\right)-{D}_{\min }\right)\times 5.0}{100}$$

The halfway time point was then determined as

$${t}_{\mathrm{halfway}}=\min \{t\in {T}_{\mathrm{search}}:\left|D\left(t\right)-{D}_{\mathrm{halfway}}\right|\le \mathrm{tolerance}\}$$

If no points fell within the tolerance range, the closest point to the halfway value was selected.

The detected halfway point was considered valid only if (1) it was not at the edge of the search window and (2) it occurred within 150 ms after fixation end. This approach provided robust detection of pattern stabilization timing while accounting for natural oscillations in MEG source dynamics.

We analyzed ventral, lateral and parietal ROI groups, followed by pooling over hemispheres. Fixation durations were divided into quartiles for comparative analysis. Differences in halfway point latency (Fig. 2e) were assessed using a linear mixed-effects model with random intercepts for participants, with fixation duration quartile, ROI and hemisphere as fixed effects and subject as a grouping variable, using ‘very short’ fixations as the reference category.

#### Ease-of-recognition analysis

We extracted image patches (100 pixels × 100 pixels; 3° of visual angle) centered on each fixation location from the stimulus scenes. These patches were processed using an AlexNet model^17^ trained on ecoset^18^, a dataset containing 565 natural-object categories. For each fixation-centered patch, we computed the entropy of the final classification layer output (565-dimensional) as an index of recognition difficulty^11^:

$$H=-\mathop{\sum }\limits_{i=1}^{565}{p}_{i}\log \left(\,{p}_{i}\right)$$

where *p*_*i*_ represents the softmax-normalized probability for class *i*. Lower entropy indicates higher classification confidence, suggesting easier recognition. We excluded last fixations from each trial and removed duration and entropy outliers per participant using the 2nd–98th percentile range. Entropy values were log-transformed to improve normality. We computed both absolute entropy (*Z*-scored per participant) and relative entropy (*Z*-scored within each scene per participant) to control for scene-level differences. For visualization, entropy values were binned into six quantiles (sextiles) per participant. The relationship between entropy measures and fixation duration was analyzed using linear mixed-effects models with the participant as a random intercept.

To verify robustness across architectures, we repeated this analysis with four additional networks (VGG16, Inception-v3, a crop-trained AlexNet variant and a Resnet50 variant). All models were trained on ecoset^18^ except the crop-trained variant, which was trained on zoomed-in ecoset images to better match the fixation crop distribution. All networks showed consistent negative relationships between classification entropy and fixation duration (Supplementary Fig. 1).

#### Caption relevance assessment

Two independent raters assessed whether fixated objects were referenced in participants’ scene descriptions from the captioning task (25% of trials). Scene captions were processed using German spaCy^58^ (‘de_core_news_sm’) for automatic noun extraction. Raters first identified spatially unspecific context nouns (for example, ‘house’ for indoor scenes) and then categorized fixation targets as self-referenced (mentioned in the participant’s caption), other-referenced (mentioned by other participants) or absent (not caption-relevant). When they selected ‘None’, raters were shown alternative nouns from other participants’ captions before final categorization. Inter-rater reliability yielded Cohen’s *κ* = 0.297 (three-way) and *κ* = 0.372 (binary self versus not-self), both indicating fair agreement with 78.0% and 81.0% raw agreement, respectively. Fixations outside the 2nd–98th percentile durations per participant and last fixations per trial were excluded. The ratings were pooled over the two raters. Mixed-effects models tested duration differences with participants as random intercepts.

#### Patch memorability analysis

We assessed the memorability of fixation targets using a deep neural network (ResMem^14^ trained on large-scale behavioral memory datasets: LaMem^19^ and MemCat^20^). For each fixation patch (100 pixels × 100 pixels; 3° of visual angle), we computed a memorability score predicting how likely it was that the visual content would be remembered in delayed recognition tests. This method provided estimates of memory processing for timescales beyond the immediate scene description task, allowing us to investigate the relationship between fixation duration and potential long-term memory encoding. We excluded the last fixation of each trial and removed fixations with durations below the second or above the 98th percentile per participant to eliminate outliers. We derived two measures per fixation: (1) absolute memorability using raw ResMem scores and (2) relative memorability (within-scene *Z*-scored ResMem scores). Both fixation durations and memorability scores were *Z*-scored within each participant to account for individual differences. We used linear mixed-effects models with random intercepts for participants to assess the relationship between memorability and fixation duration. For visualization (Fig. 3d), memorability scores were divided over six quantiles (sextiles) per participant and subsequently averaged. To verify that viewing time did not substantially influence fixation durations, we examined fixation duration as a function of time in trial (Supplementary Fig. 3). Following an initial increase from the first fixation, fixation durations remained relatively stable throughout the exploration sequence.

To test whether the memorability effect was confounded by upcoming saccade planning, we matched each fixation to its subsequent saccade and computed saccade amplitude as the Euclidean distance between fixation end and saccade end coordinates (in pixels). We included saccade amplitude and its interaction with memorability as predictors in a mixed linear model with subject as random effect. Both amplitude and memorability scores were *Z*-scored per subject before analysis.

#### Semantic category analysis

To examine whether the memorability effect generalized across semantic content, we classified fixation targets into semantic categories using a VGG16 model trained on ecoset (565 object categories). For each fixation-centered crop (100 pixels × 100 pixels), we extracted final classification layer activations and assigned the predicted category based on the maximum softmax probability. Classifications with confidence below the fifth percentile were excluded. Fixation targets were grouped into animate (human, animal) and inanimate (natural, manmade) categories based on ecoset’s semantic taxonomy. Separate linear mixed-effects models (with random intercepts per participant) were fitted for animate and inanimate targets to test whether the memorability effect persisted across both semantic categories (Supplementary Fig. 2).

#### Decoding analyses

To provide neural validation, we decoded ease-of-recognition and memorability estimates from gradiometer patterns using ridge regression. As linear decoders are sensitive to outlier data points, epochs were excluded if (1) maximum amplitude exceeded the 99th percentile or (2) correlation with the median ERF fell below the first percentile (computed per participant on flattened channel × time data).

#### Ease-of-recognition decoding

We decoded AlexNet–ecoset classification entropy values from gradiometer patterns. Following quality control, data were preprocessed using robust scaling (clipping outliers beyond 5 s.d. per channel and session). We used fivefold cross-validation with scene-based grouping and a sliding estimator approach (ridge regression at each time point from −200 ms to +500 ms post-fixation). Decoder performance (*R*^2^) was compared against dummy baseline (mean-prediction) decoders using paired *t*-tests (Fig. 2h). Decoded entropy values were then used to predict fixation durations, testing whether MEG-decoded recognition difficulty preserved the inverse relationship with fixation duration (Fig. 2i).

#### Memorability decoding

The same pipeline was applied to decode ResMem memorability scores (both absolute and relative within-scene *Z*-scored values; Fig. 3e).

#### PAC analysis

To examine neural signatures associated with memory encoding during longer fixations, we analyzed PAC between theta (3–8 Hz) and gamma (40–140 Hz) oscillations using source-reconstructed MEG data. To isolate oscillatory activity from evoked responses, we implemented a fourfold median ERF subtraction procedure. For each epoch, we subtracted (1) the median saccade-onset ERF, (2) the median fixation-onset ERF, (3) the median ERF of the subsequent saccade and (4) the median ERF of the subsequent fixation. This fourfold residual approach removed both onset-locked and offset-locked evoked components while preserving oscillatory dynamics. Mirroring the decoding analyses, epochs were excluded if (1) their maximum amplitude exceeded the 99th percentile or (2) correlation with the median ERF fell below the first percentile (computed per participant on flattened channel × time data).

Source-reconstructed data were filtered using causal minimum-phase FIR filters:

$$\begin{array}{l}{x}_{\theta}(t) = {\mathrm{FIR}}_{{\mathrm{min}}{\mathrm{-}}{\rm{phase}}}[x(t),\, {3\,{\rm{Hz}}},\, {8 \,{\rm{Hz}}}] \\ {x}_{\gamma}(t) = {\mathrm{FIR}}_{{\mathrm{min}}{\mathrm{-}}{\rm{phase}}}[x(t),\, {40\,{\rm{Hz}}},\, {140\, {\rm{Hz}}}]\end{array}$$

With Hilbert transform, we extracted instantaneous phase (theta) and amplitude (gamma):

$${\phi}_{\theta}\left(t\right) = \angle\left[\mathrm{H}\left({x}_{\theta}\left(t\right)\right)\right]$$

$${A}_{\gamma}\left(t\right) = \left|\mathrm{H}\left({x}_{\gamma}\left(t\right)\right)\right|$$

Phase and amplitude estimation via Hilbert transform was performed on the full epoch length (−500 ms to +800 ms relative to fixation onset; 1,300 ms total) to ensure reliable frequency decomposition, particularly for low-frequency theta oscillations (for example, ~3 Hz, requiring ~330 ms per cycle). Following phase/amplitude extraction, analyses were restricted to specific time windows as detailed below. For direct comparison between fixation durations, we employed an offset-locked approach. Rather than using fixed time windows relative to fixation onset, we extracted PAC windows relative to fixation end (offset). For both longer (>300 ms) and shorter (<300 ms) fixations, we analyzed a 250-ms window ending at fixation offset. Given that shorter fixations may not provide sufficient preoffset data, we permitted the analysis window to extend up to 75 ms beyond the fixation offset (duration + 75 ms postoffset ≥ 250 ms window requirement). This extension into the early postoffset period was deemed acceptable because (1) the fourfold median ERF subtraction procedure removed saccade-related evoked activity (Figs. 4a) and (2) both duration groups were subjected to the same temporal extension, maintaining comparability. To ensure equal numbers of epochs per condition and control for potential sampling biases, we implemented duration-balanced sampling by randomly selecting equal numbers of longer and shorter fixations per participant.

PAC was computed using the modulation index method following ref. ^16^. For each phase bin *j*, the mean gamma amplitude was calculated:

$${\bar{A}}_{j}=\frac{1}{\left|{I}_{j}\right|}\sum_{i\in {I}_{j}}{A}_{\gamma}\left({t}_{i}\right)$$

where ${I}_{j}=\left\{i:{\phi}_{\mathrm{bin},\,j-1}\le {\phi}_{\theta}\left({t}_{i}\right) < {\phi}_{\mathrm{bin},\,j}\right\}$ for *N* = 18 equally spaced phase bins from −*π* to *π*. The binned amplitudes were normalized to form a probability distribution:

$${p}_{j}=\frac{{\bar{A}}_{j}+\epsilon}{\sum_{k=1}^{N}({\bar{A}}_{k}+\epsilon)}$$

where $\epsilon ={10}^{-10}$ prevents division by zero. The modulation index was then computed as the normalized Kullback–Leibler divergence:

$$\mathrm{MI}=\frac{1}{\log N}\sum_{j=1}^{N}{p}_{\!j}\log\left(N{p}_{\!j}\right)$$

Statistical significance was assessed using 200 surrogate samples generated with the single-cut method, which randomly cuts each epoch’s time series and swaps segments to preserve temporal structure while disrupting phase–amplitude relationships:

$${\phi }_{\theta }^{\left(b\right)}\left(t\right)={\phi }_{\theta }\left(t+{c}_{b}\right)\,{\tt{if}}\, {t}+{c}_{b}\le T$$

or

$${\phi }_{\theta }^{\left(b\right)}\left(t\right)={\phi }_{\theta }\left(t+{c}_{b}-T\,\right)\,{\tt{if}}\,{t}+{c}_{b} > T$$

where *c*_*b*_ is a random cut point for bootstrap iteration *b*. PAC *Z*-scores were computed against the surrogate distribution:

$$z=\frac{{\mathrm{PAC}}_{\mathrm{observed}}-{\mu }_{\mathrm{surrogate}}}{{\sigma }_{\mathrm{surrogate}}}$$

PAC values from all vertices within each anatomical label were pooled across participants and hemispheres. *Z*-scores were converted to *P* values, and false discovery rate (Benjamini–Hochberg) correction (*P* < 0.001) was applied across all vertices within each participant to control for multiple comparisons. A mixed-effects linear model with participant as a random effect was fitted to compare PAC *Z*-scores across brain areas.

A separate frequency-by-frequency analysis examined coupling across extended frequency ranges: theta (1–15 Hz) and gamma (40–190 Hz). For this analysis, phase filters used fixed 2-Hz bandwidths (±1 Hz around center frequency) stepped in 1-Hz increments. Following Aru et al.^15^, amplitude filters typically use bandwidths equal to twice the center phase frequency to maintain appropriate frequency resolution. However, to minimize potential spectral leakage from the phase frequency into the amplitude frequency range, a concern that arises especially when phase and amplitude bands are in close proximity^16,59^, we employed a more conservative approach, using bandwidths equal to four times the center phase frequency (4 × frequency for phase). Amplitude frequencies were sampled in 5-Hz steps to provide fine resolution across the gamma range. The resulting PAC matrices were smoothed using bicubic interpolation for visualization.

### Reporting summary

Further information on research design is available in the Nature Portfolio Reporting Summary linked to this article.

## Online content

Any methods, additional references, Nature Portfolio reporting summaries, source data, extended data, supplementary information, acknowledgements, peer review information; details of author contributions and competing interests; and statements of data and code availability are available at 10.1038/s41593-026-02285-1.

## Supplementary information

Supplementary Information Supplementary Figs. 1–4.

Reporting Summary

Supplementary Data 1 Source data for supplementary figures.

## Source data

Source Data Statistical Source Data for Figs. 1–4.

## Data Availability

All derived data necessary to reproduce the analyses and figures are available via GRO.data at 10.25625/DDJ5C3 (ref. ^60^). Source data are provided with this paper.

## Code availability

The complete analysis code is publicly available via GitHub at https://github.com/KietzmannLab/memdur.
