# Supplementary material for: Fixation duration on natural scenes is explained by memory encoding not processing demand
Source: Nat Neurosci. 2026 May 25;29(6):1488–97. doi: 10.1038/s41593-026-02285-1 (PMC13246442; doi:10.1038/s41593-026-02285-1)
Supplement: Supplementary file 1 — Supplementary Figs. 1–4. [file 41593_2026_2285_MOESM1_ESM.pdf]

nature neuroscience

Supplementary information https://doi.org/10.1038/s41593-026-02285-1

Fixation duration on natural scenes is explained by memory encoding not processing demand

In the format provided by the authors and unedited

Supplementary material for NN-A91421A

A Processing demand hypothesis model comparison

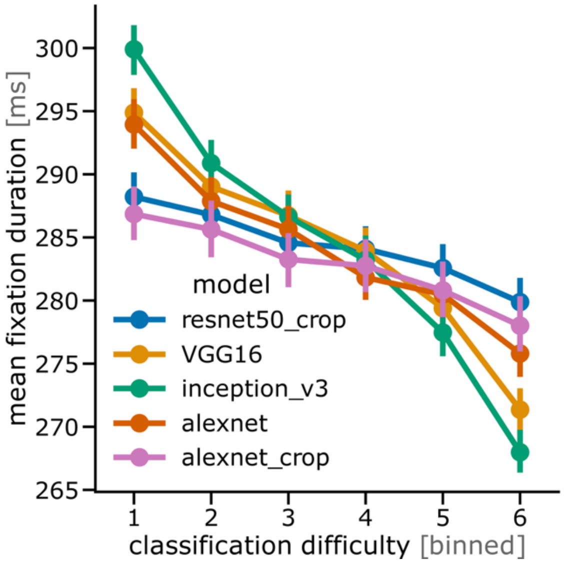

Supplementary Figure 1 | Classification difficulty predicts fixation duration across network architectures. Mean fixation duration as a function of classification entropy binned into sextiles, shown for five convolutional neural network architectures. All models were trained on ecoset (565 object categories), except alexnet_crop and resnet50_crop which were trained on zoomed-in/cropped ecoset images. Lower entropy (bin 1) indicates easier recognition, higher entropy (bin 6) indicates more difficult recognition. All architectures show consistent negative relationships between classification difficulty and fixation duration (all $p < .001$), with easier-to-recognise content receiving longer fixations. 5 participants; 1,515,835 total fixations across 5 architectures Error bars show 95% confidence intervals. Entropy values were log-transformed and z-scored per participant (absolute entropy shown). The inverse relationship between recognition difficulty and viewing time is robust across model architectures.

A Memory facilitation hypothesis: Category effects

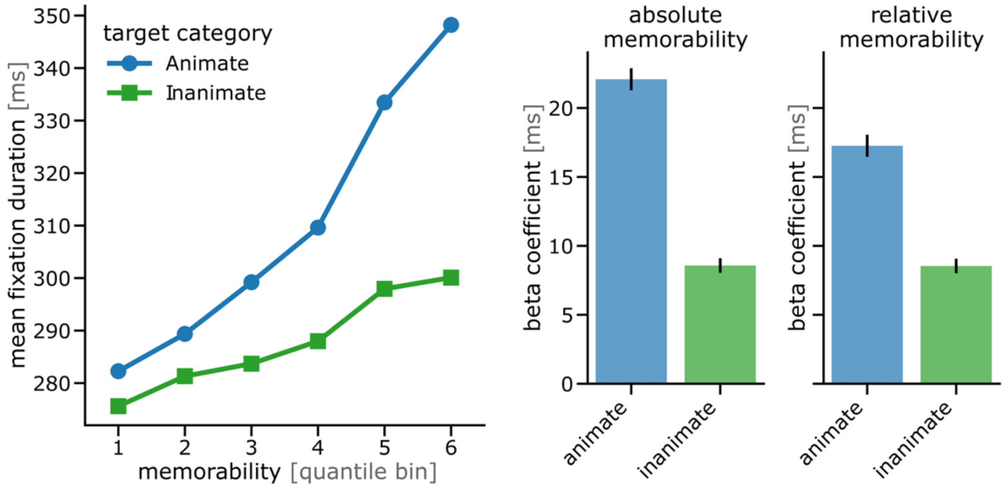

Supplementary Figure 2 | Memorability effects persist across semantic categories. A) *Left*: Mean fixation duration as a function of memorability (binned into sextiles) shown separately for animate (blue; n=52,894 fixations) and inanimate (green; n=95,764 fixations) targets, based on VGG16 ecoset classifications. Both categories show positive relationships between memorability and fixation duration. *Middle* and *right*: Beta coefficients from linear mixed-effects models for absolute memorability (z-scored per participant) and relative memorability (z-scored per scene). The memorability effect was significant for both animate targets (absolute: β=22.09ms, 95% CI [20.53, 23.65], $p < .001$; relative: β=17.26ms, 95% CI [15.69, 18.84], $p < .001$) and inanimate targets (absolute: β=8.58ms, 95% CI [7.54, 9.61], $p < .001$; relative: β=8.54ms, 95% CI [7.51, 9.58], $p < .001$), demonstrating that, albeit being stronger in the animate category, the memorability effect occurs across semantic categories. Error bars show 99% confidence intervals.

A Viewing time and fixation duration

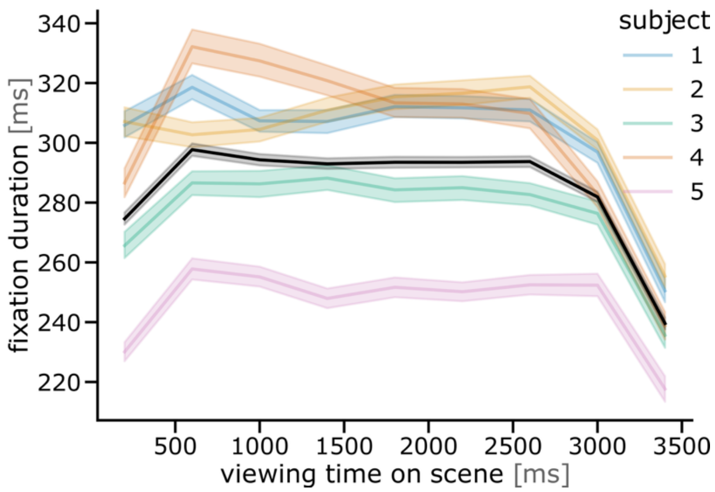

Supplementary Figure 3 | Fixation duration across exploration sequence. A) Mean fixation duration as a function of viewing time, shown separately for each subject (coloured lines, with shaded regions indicating standard (95% CIs). 5 participants; 163,896 fixations binned into 400 ms intervals. Following an initial increase from the first fixation, fixation durations remained relatively stable throughout the exploration sequence. The apparent decrease towards the end of viewing (after approximately 3000 ms) likely reflects survivorship bias, as only fixations completed on the scene are included. The black line shows the group average with 95%CI.

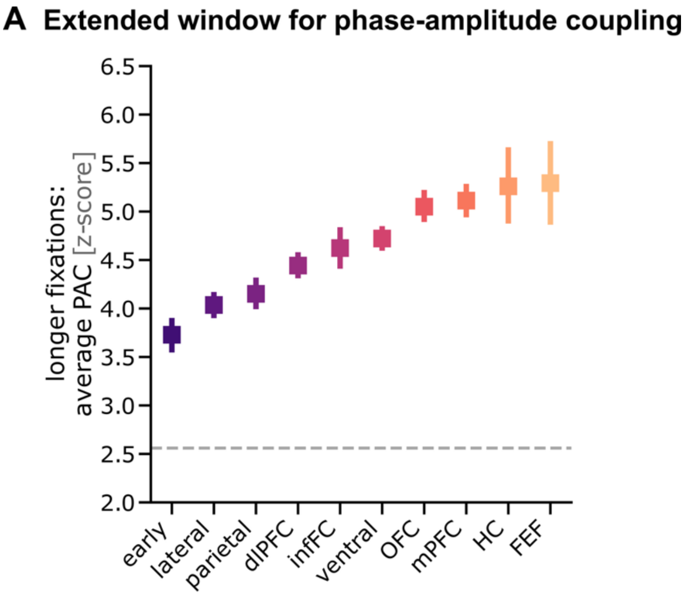

Supplementary Figure 4 | Extended window phase-amplitude coupling analysis A) Mean theta-gamma phase-amplitude coupling (PAC) strength for longer fixations (>350 ms) across brain regions, computed using an extended offset-locked window (final 350 ms before fixation end). 5 participants; 10 participant-hemisphere observations across 10 ROIs. Error bars: bootstrapped 95% CIs over participant-hemisphere ROI means. Dashed line: $z = 2.56$ ($p < 0.001$). The grey dashed line indicates the significance threshold ($z = 2.56$, $p < .001$). PAC remains significantly elevated above baseline across all regions, with strongest coupling in frontal areas. This extended window analysis indicates that the duration-dependent PAC effects are robust to the specific temporal window used for analysis.
