# Supplementary material for: Fixation duration on natural scenes is explained by memory encoding not processing demand
Source: Nat Neurosci. 2026 May 25;29(6):1488–97. doi: 10.1038/s41593-026-02285-1 (PMC13246442; doi:10.1038/s41593-026-02285-1)
Supplement: Supplementary file 3 — Source data for supplementary figures. [file 41593_2026_2285_MOESM3_ESM.zip › supplement_source_data/Supplementary figure source data - table of contents.docx]

| Type | Number  Each type of file (Table, Video, etc.) should be numbered from 1 onwards. Multiple files of the same type should be listed in sequence, i.e.: Supplementary Video 1, Supplementary Video 2, etc. | Filename  Whole original file name including extension. i.e.: *Smith_ Supplementary_Video_1.mov* | Legend or Descriptive Caption  Describe the contents of the file |
| --- | --- | --- | --- |
| Supplementary Data | 1 | sourcedata_network_comparison_quartiles.csv | Source Data for Supplementary Figure 1: mean fixation duration by classification entropy sextile for five convolutional neural network architectures (AlexNet, VGG16, Inception-v3, AlexNet-crop, ResNet50-crop) |
| Supplementary Data | 2 | sourcedata_memorability_by_animacy_quartiles.csv | Source Data for Supplementary Figure 2: fixation duration by memorability sextile, separately for animate and inanimate fixation targets |
| Supplementary Data | 3 | sourcedata_duration_by_viewing_time.csv | Source Data for Supplementary Figure 3: mean fixation duration per 400 ms viewing time bin across the exploration sequence |
| Supplementary Data | 4 | sourcedata_fig4c_pac_per_roi.csv | Source Data for Supplementary Figure 4: mean PAC z-score per ROI for longer fixations (extended 350 ms window analysis) |

***Add rows as needed to accommodate the number of files.***
