# Supplementary material for: Fixation duration on natural scenes is explained by memory encoding not processing demand
Source: Nat Neurosci. 2026 May 25;29(6):1488–97. doi: 10.1038/s41593-026-02285-1 (PMC13246442; doi:10.1038/s41593-026-02285-1)
Supplement: Supplementary file 4 — Statistical Source Data for Figs. 1–4. [file 41593_2026_2285_MOESM4_ESM.zip › source_data/manuscript/Source data table of contents.docx]

| Parent Figure or Table | Filename  Whole original file name including extension. i.e.: *Smith_SourceData_Fig1.xls,* or *Smith_ Unmodified_Gels_Fig1.pdf* | Data description  i.e.: Unprocessed western Blots and/or gels, Statistical Source Data, etc. |
| --- | --- | --- |
| Source Data Fig. 1a | sourcedata_quartile_distribution_filt50Hz_bilateral.csv | Statistical Source Data: fixation duration distribution by quartile |
| Source Data Fig. 1c | sourcedata_fig1c_tsne_clusters.csv | Statistical Source Data: t-SNE coordinates and cluster assignments for all scenes |
| Source Data Fig. 1d | sourcedata_fig1d_cluster_share_avs_nsd.csv | Statistical Source Data: AVS vs NSD cluster share proportions |
| Source Data Fig. 2a | sourcedata_quartile_distribution_filt50Hz_bilateral.csv | Statistical Source Data: fixation duration distribution by quartile |
| Source Data Fig. 2d | sourcedata_dynamics_(mid)ventral_filt50Hz_bilateral.csv | Statistical Source Data: ventral cortex MEG pattern change dynamics by duration quartile |
| Source Data Fig. 2e left | sourcedata_duration_by_quartile_filt50Hz_bilateral.csv | Statistical Source Data: mean fixation duration per quartile |
| Source Data Fig. 2e right | sourcedata_t_halfway_by_roi_quartile_filt50Hz_bilateral.csv | Statistical Source Data: post-peak halfway latency per ROI × quartile |
| Source Data Fig. 2g | sourcedata_all_subjects_100px_crops_entropy_log_activation_quartile_plot.csv | Statistical Source Data: fixation duration by classification entropy sextile |
| Source Data Fig. 2h | sourcedata_entropy_timepoint_decoding_grad.csv; sourcedata_entropy_timepoint_decoding_baseline_grad.csv | Statistical Source Data: entropy decoding R² over time (real and baseline decoders) |
| Source Data Fig. 2i | sourcedata_duration_per_predicted_entropy_quartile_entropy.csv; sourcedata_duration_per_predicted_entropy_quartile_entropy_relative.csv | Statistical Source Data: fixation duration by MEG-predicted entropy sextile (absolute and relative) |
| Source Data Fig. 3b | sourcedata_fix2cap_duration_by_condition_all.csv | Statistical Source Data: fixation duration by caption-mention condition |
| Source Data Fig. 3c | sourcedata_memorability_histogram.csv | Statistical Source Data: memorability score distribution per subject |
| Source Data Fig. 3d | sourcedata_duration_per_memorability_quartile.csv | Statistical Source Data: fixation duration by memorability sextile |
| Source Data Fig. 3e left | sourcedata_memorability_timepoint_decoding_mag.csv; sourcedata_memorability_timepoint_decoding_baseline_mag.csv | Statistical Source Data: memorability decoding R² over time (real and baseline decoders) |
| Source Data Fig. 3e right | sourcedata_duration_per_predicted_mem_quartile_memorability.csv; sourcedata_duration_per_predicted_mem_quartile_mem_relative.csv | Statistical Source Data: fixation duration by MEG-predicted memorability sextile (absolute and relative) |
| Source Data Fig. 4c | sourcedata_fig4c_pac_per_roi.csv | Statistical Source Data: mean PAC z-score per ROI (longer fixations) |
| Source Data Fig. 4d | sourcedata_pac_HC_lh_long_dur_smooth_False.csv; sourcedata_pac_HC_lh_long_dur_smooth_True.csv | Statistical Source Data: frequency-by-frequency PAC matrix for HC left hemisphere (raw and smoothed) |
| Source Data Extended Data Fig./Table 10 |  |  |
