# Supplementary figures and images for: Fixation duration on natural scenes is explained by memory encoding not processing demand
Source: Nat Neurosci. 2026 May 25;29(6):1488–97. doi: 10.1038/s41593-026-02285-1 (PMC13246442; doi:10.1038/s41593-026-02285-1)

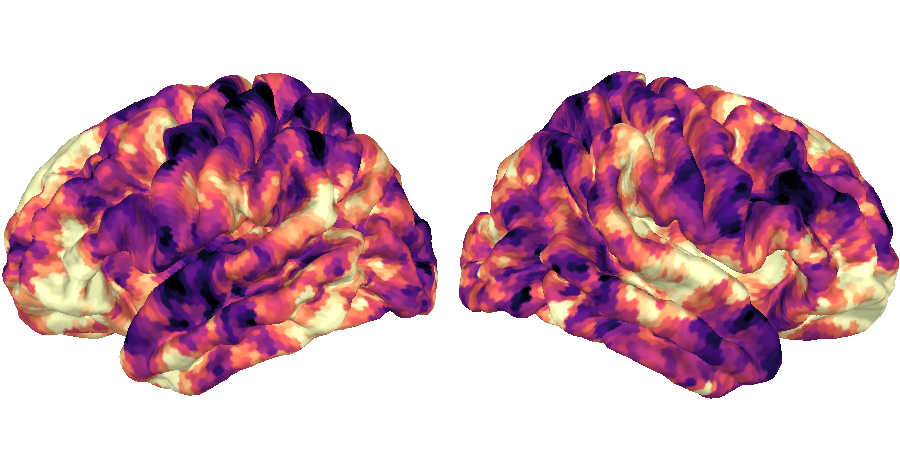

Supplement: Supplementary file 4 — Statistical Source Data for Figs. 1–4. [file 41593_2026_2285_MOESM4_ESM.zip › source_data/manuscript/fig4/fig4b/pac_average_saccade_3-8_40-140_pial_lateral.png]

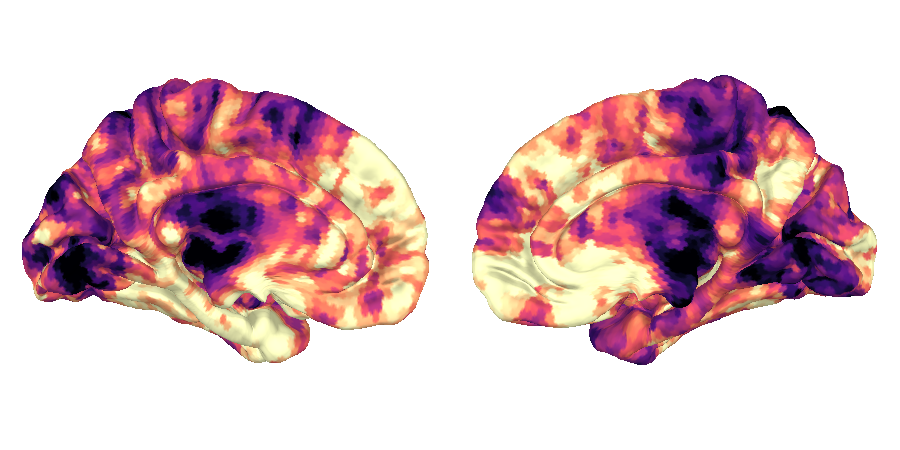

Supplement: Supplementary file 4 — Statistical Source Data for Figs. 1–4. [file 41593_2026_2285_MOESM4_ESM.zip › source_data/manuscript/fig4/fig4b/pac_average_saccade_3-8_40-140_pial_medial.png]
